# Taxonomy, Phylogeny, Genomes, and Repeatomes in the Subgenera *Salvia*, *Sclarea*, and *Glutinaria* (*Salvia*, Lamiaceae)

**DOI:** 10.3390/ijms26136436

**Published:** 2025-07-04

**Authors:** Julia V. Kalnyuk, Olga Yu. Yurkevich, Ekaterina D. Badaeva, Alexey R. Semenov, Svyatoslav A. Zoshchuk, Alexandra V. Amosova, Olga V. Muravenko

**Affiliations:** Engelhardt Institute of Molecular Biology, Russian Academy of Sciences, 32 Vavilov St, 119991 Moscow, Russia

**Keywords:** *Salvia*, *Sclarea*, *Glutinaria*, genome, repeatome, chromosome, rDNAs, NGS, satDNAs, FISH

## Abstract

The genus *Salvia* L. (Lamiaceae) is characterized by complex taxonomy and controversial phylogeny. This genus includes about a thousand species with worldwide distribution and high ecological, structural, functional and morphological diversity. Because of their high content of essential oils, various *Salvia* plants are widely used in medicine, as well as in the food, perfume, cosmetic, and paint industries; they also are valuable melliferous resources. The present study reviews the taxonomic history of the genus *Salvia* and the phylogenetic relationships between the taxa within the subgenera *Salvia*, *Sclarea*, and *Glutinaria*. Among the *Salvia* species, three basic chromosome numbers, *x* = 7, *x* = 8, and *x* = 11, were most common, although other basic chromosome numbers (*x* = 6–19) were determined, which was probably due to events of dysploidy, aneupoidy, and/or polyploidy occurring during speciation. Recent molecular cytogenetic studies based on Next Generation Sequencing technologies have clarified the chromosomal organization of several *Salvia* species. The patterns of chromosome distribution of 45S rDNA, 5S rDNA, and satellite DNAs made it possible to assess their intra- and interspecific chromosome diversity. However, further cytogenetic studies are needed to characterize the chromosomes in the genomes of other *Salvia* species and specify the genomic relationships among them.

## 1. Introduction

The genus *Salvia* L. (Lamiaceae) includes about a thousand species widely distributed worldwide [1,2]. *Salvia* organisms are mainly represented by herbaceous plants and shrubs, but in the subg. *Calosphace*, woody plants are also described [3]. Because of their high content of essential oils, various *Salvia* plants are widely used in medicine, as well as in the food, perfume, cosmetic, and paint industries; they are also valuable melliferous resources [4,5]. Many *Salvia* species contain biologically important compounds, such as alkaloids, flavonoids, terpenes and terpenoids, phenylpropanoids, phenolic acids, glycoside derivatives, and lignans [4,6,7]. These compounds demonstrate a wide spectrum of pharmacological activities, including antibacterial, fungicidal, anti-inflammatory, antioxidant, immunostimulant, and antitumor effects [4,5,8]. In addition, hypoglycemic and hypolipidemic effects and properties that improve cognitive function have been described in *Salvia* species [5,6,7]. *S. sclarea*, *S. officinalis*, *S. pratensis*, *S. nutans*, *S. nemorosa*, *S. viridis*, *S. multihrrisa*, and *S. glutinosa*, are also used as ornamental crops [3,9].

The genus *Salvia* is characterized by a complex taxonomy and a controversial phylogeny [1,2,9]. The first morphological classification was proposed by Bentham [10], who divided the genus *Salvia* into 12 sections based on the peculiar lever-like stamen structure inherent to this genus. Subsequent attempts to revise the traditional morphology-based classifications resulted in different variants of the genus taxonomy [11,12,13]. Molecular phylogenetic studies demonstrated the polyphyletic origin of the genus *Salvia*, which resulted in other variants of its taxonomy [14,15,16]. Because of the high morphological and genetic diversity within *Salvia* [1,9,17] a unified approach to its taxonomy has not yet been developed, and further molecular genetic studies are needed to clarify the taxonomy of this genus.

Among the species within the genus *Salvia*, high variability of basic chromosome numbers (from 6 to 19) was revealed [18]. Genome sizes can vary greatly among the species, and they do not always correlate with the number of chromosomes in the karyotypes [19]. In addition, chromosome sizes in *Salvia* species are quite small, which makes it difficult to conduct comparative genomic studies. The use of tandem DNA repeats as chromosomal markers has provided insights into the evolution of some valuable sage species [20,21,22]. Moreover, high-throughput sequencing of the genomes and transcriptomes of various *Salvia* species [23,24,25,26,27,28] has opened up new opportunities to identify evolutionarily significant events of full-genome duplications, clarify the phylogenetic relationships of the species genomes, study the chromosomal structure in karyotypes using tandem DNAs, and refine the evolutionary pathways of different taxa within the genus.

**Figure 1:**
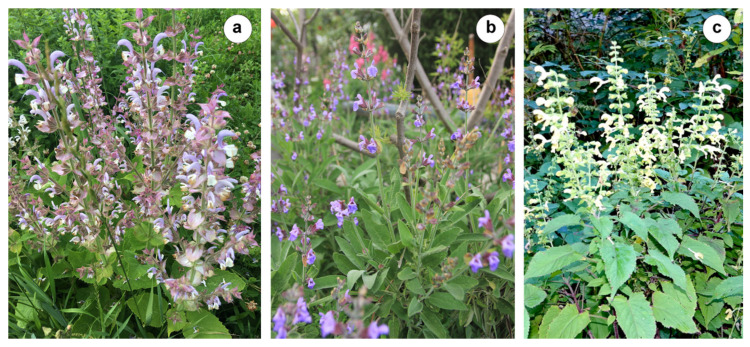
Representative species from the subgenera Salvia, Sclarea, and Glutinaria: (**a**) *Salvia sclarea* subg. Sclarea (Moscow region, Russia); (**b**) *Salvia officinalis* subg. Salvia (Novorossiysk, Russia); (**c**) *Salvia glutinosa* subg. Glutinaria (Akamara village, Republic of Abkhazia). The images were taken by O. Yurkevich (**a**), M. Luchkin CC BY 2025 (**b**), and L. Saplitskaya (**c**).

In this paper, we reviewed current *Salvia* taxonomic and phylogenetic studies, as well as the genome and repeatome peculiarities in economically valuable species of the subgenera *Glutinaria*, *Salvia*, and *Sclarea* of the genus *Salvia* (Figure 1).

## 2. Taxonomy and Phylogeny of the Genus Salvia

According to different databases of vascular plants, the genus *Salvia* contains 1036 [29] or 1045 [30] accepted species. This genus is characterized by worldwide distribution and high ecological, structural, functional, and morphological diversity [31,32,33]. In this regard, the morphology, function, and evolution of floral traits in *Salvia* are of particular interest. *Salvia* species are morphologically characterized by unusual lever-like stamens formed by elongated connective and stamen filaments [16,34], which could contribute to saving and dispensing of pollen. This staminal lever mechanism is a key innovation in *Salvia.* Because of this staminal lever mechanism and floral structures, complete emptying of the pollen sacs occurs, which increases the rate of out-crossing and forces diversification and speciation within this genus (Table 1) [34]. The morphology, function, and diversity of the lever-like stamens, including the absence vs. presence of versatile anthers, bithecate vs. monothecate anthers, curved vs. straight connective growth, and the presence vs. absence of the lower lever arm, are important floral traits in *Salvia* to understand the lever mechanism and to reconstruct the evolution of the stamen. This special lever-like pollination mechanism could play an important role in the success of pollination in *Salvia*, as well as in the diversification of the genus [2,34,35,36,37,38]. Moreover, based on the lever-like pollination mechanism, a cladogram with detailed descriptions and images of different stamen types (A–N) found in each clade was constructed (detailed in Table 1) [37,39].

Based on the morphology of the calyx, corolla, and stamens, as well as on the geographical distribution of *Salvia* species, this genus was divided into twelve sections [10], which were classified into four subgenera [38]. According to this classification, the subg. *Salvia* included the sections *Hymenosphace*, *Drymosphace*, and *Eusphace*, and the subg. *Sclarea* contained the sections *Horminum*, *Aethiopis*, and *Plethiosphace*. Briquet [11] subsequently presented a revised classification of this genus, identifying eight subgenera (*Salvia*, *Schraderia*, *Allagospadonopsis*, *Sclarea*, *Leonia*, *Viasala*, *Covola*, and *Jungia*) and seventeen sections. These two comprehensive classifications were further modified by various authors. In particular, Hedge [12] believed that the classifications of Bentham and Briquet were outdated and that there were species that could not be classified into any of these subgenera. He believed that groups of species that had morphological similarities, and sometimes the same geographic distribution, were the most natural supraspecific taxa, and accordingly, he divided 59 African species of *Salvia* into 23 species groups. At the same time, in Flora of the USSR [13], *Salvia* is divided into seven subgenera: subg. *Eusalvia*, subg. *Macrosphace*, subg. *Leonia*, subg. *Sclerea*, subg. *Jangia*, subg. *Covola*, and subg. *Sanglakia*; the subg. *Schraderia* is placed in a separate genus. Thus, the peculiarities of the stamen structure and the diversity of the flower morphology in different *Salvia* species have resulted in different variants of intrageneric classification, and a generally accepted taxonomy of the genus *Salvia* based on only morphological characters was not developed [2,9,14,15,39].

The morphological diversity within the genus *Salvia* is consistent with a high level of genetic variability revealed by studying the genomes of *Salvia* species using various molecular genetic approaches [40]. Studying the genomes of *Salvia* species using DNA markers, including rDNA internal transcribed spacer (ITS) regions, chloroplast intergenic spacers, RAPD, ISSR and AFLP, microsatellite markers (SSRs), sequence-related amplified polymorphism (SRAP), polymerase chain reaction–restriction fragment length polymorphism (PCR-RFLP), conserved region amplification polymorphism (CoRAP), and directed amplification of minisatellite DNA region (DAMD-PCR), made it possible to clarify the taxonomic and phylogenetic relationships between and within the species, as well as to find markers for gene identification [40]. In particular, significant intra- and interpopulation genetic polymorphism was detected within 25 geographic populations of the perennial species *S. officinalis* using AFLP DNA markers [41]. The level of genetic diversity was assessed in 15 species of the genus *Salvia* using multilocus DNA markers, ISSR and RAPD, which demonstrated high intra- and interspecific genetic differentiation within the genus [42,43,44]. The analysis of the relationship and genetic variability among *Salvia* species using cross-amplification of microsatellite DNA markers (SSR) made it possible to estimate the evolutionary distances between them [45,46]. Thus, investigation of genetic diversity in species from the genus *Salvia* contributed to the clarification of their phylogenetic relationships. At the same time, the origin of this genus remained controversial for a long time.

The genus *Salvia* was long considered to be monophyletic based on the unusual structure of the stamens and the assumption that their specific lever-like mechanism evolved once in the tribe Mentheae [14,32]. At the same time, this mechanism is considered to be a unique morphological feature, and even a minor change in the lever-like stamens could lead to evolution in groups as well as radiation of species within the genus *Salvia* [32,39]. In more recent studies, five types of stamens were described, and the distribution of stamen types within the genus demonstrated that the stamen linkage evolved in parallel at least three times, supporting a polyphyletic origin of this genus [37]. At present, no evidence has been found to directly link the lever mechanism of the stamen structure with the divergence of *Salvia* species [47]. Taxonomists believe that in order to compile a more accurate classification of *Salvia* species, one should rely not only on the morphology of the stamens but on other floral traits (for example, the corolla length as a diversification feature) [47].

Molecular phylogenetic studies of *Salvia* species using the chloroplast DNA regions *rbcL* and *trnL-F* as markers have refuted the monophyletic origin of the genus. It was established that the stamen lever mechanism evolved in parallel at least twice, and three branches of evolution (three clades) of species of this genus were identified. Clade I included mostly the Old-World species and one New-World lineage. Clade II, which comprised the subgroup *Calosphace* and the section *Audibertia*, was an evolutionary lineage from the New World. Clade III represented an independent Asian line [14]. Based on nrITS analysis, four distinct evolutionary lineages (four clades) were identified in 220 *Salvia* species [9]. Comparative studies showed that 15 species from the genera related to *Salvia* (*Dorystaechas* (Boiss. & Heldr. ex Benth.), *Meriandra* (Benth.), *Perovskia* (Kar.), *Rosmarinus* L., and *Zhumeria* (Rech. f. & Wendelbo)) should be included in the genus *Salvia* [1]. However, the stamen structure in most species from these five genera differed from the specific lever-like stamen mechanism observed in *Salvia* [1,2,14,16].

Based on the results of molecular studies, the taxonomy of the genus *Salvia* was also revised [1,15]. Some taxonomists believe that each subgenus should be separated into a certain genus. They divided the genus *Salvia* into 11 subgenera, leaving the name ‘*Salvia*’ for the subgenus to which *S. officinalis* belongs [9,15]. Other researchers considered that such a division would lead to confusion, since a great number of species would have to be renamed [1]. Currently, most taxonomists support the widespread classification of *Salvia*, that was developed based on the results of both morphological and molecular phylogenetic studies [1,2,16,48]. According to this classification, the genus *Salvia* is divided into 11 subgenera, which form three clades [1,2]. Clade I includes the subgenera *Salvia*, *Sclarea*, and *Heterosphace*. It mainly comprises species from southwest Asia and the Mediterranean, southern and eastern Africa, Madagascar, and Central America. The subgenera *Perovskia* and *Rosmarinus* (both previously recognized as genera) represent early divergent branches of clade I. Clade II (subg. *Glutinaria*) is almost exclusively East Asian, with several species being more widespread in central and western Eurasia. Clade III comprises the subgenera *Zhumeria* and *Dorystaechas* (the Old World), the subg. *Meriandra* (formerly separate genus), and the subgenera *Audibertia* and *Calosphace* (the New World) [1,2,16].

Recent studies using high-throughput sequencing of genomes and transcriptomes of *Salvia* species have greatly clarified the origins of the species, subgenera, and genus [16,48,49]. The results obtained confirmed both the polyphyletic origin of this genus and the monophyletic origin of its individual subgenera or groups of subgenera. *Glutinaria* was found to be a monophyletic subgenus, despite the fact that its species are characterized by diverse stamen types and were previously classified into three separate subgenera (*Salvia*, *Sclarea*, and former *Allagospadonopsis*) [16]. The subgenera *Rosmarinus*, *Perovskia*, *Heterosphace*, *Salvia*, and *Sclarea* were shown to be sister clades. Monophyly was confirmed within the subgenera *Heterosphace*, *Salvia*, and *Sclarea*, and close relationships were established between the subgenera *Salvia* and *Sclarea* [48]. Phylogenetic analysis using chloroplast genomes from GenBank showed that plastome genetic characteristics were associated with plant geographic distribution, and *S. rosmarinus* was grouped with *S. officinalis* and *S. sclarea*, indicating their close relationship [49]. The monophyletic origin of the subg. *Audibertia* still remains questionable and requires further clarification [48]. The investigation of the single-copy nuclear loci, as well as sequencing of the complete plastome and mitochondrial sequences, provided additional information that helped to assess the degree of relationship of species and clarify their systematic position within the genus *Salvia* [50,51,52,53]. At the same time, the taxonomy of *Salvia* is still considered imperfect, and further studies of *Salvia* species genomes are needed to develop more accurate classification within this genus.

## 3. Genomes and Repeatomes in the Subgenera Salvia, Sclarea, and Glutinaria

### 3.1. Karyological Studies of the Salvia Species

For the first time, the term “genome” was proposed by Winkler in 1920 to describe the haploid chromosome set [54]. Currently, the haploid chromosome set in plant gametophyte cells is called ‘the basic chromosome number’ and considered a taxonomic feature [55]. Among the taxa of the genus *Salvia*, a wide range of chromosome numbers (*x* = 6, 7, 8, 9, 10, 11, 13, 14, 15, 16, 17, and 19) have been found [56,57,58,59,60,61]. Such chromosome number diversity could be related to events of interspecific hybridization found in *Salvia* [18]. It was shown that closely related species of *Salvia* could hybridize successfully [62,63,64]. Moreover, there is evidence on interspecific hybrids of plants from different sections of this genus [65,66].

Furthermore, according to chromosome databases, B chromosomes (Bs) were revealed in the karyotypes of many *Salvia* species [67], and to some extent, the presence of these extra chromosomes may explain the variability in chromosome numbers [61]. Bs are dispensable supernumerary genomic components that have been revealed in the genomes of many eukaryotes. Bs can be inherited but do not follow Mendelian rules. Moreover, Bs can be spontaneously generated in response to new genomic states that occur, for example, after interspecific hybridization events [68].

In the different geographical locations of *Salvia* species, the most common chromosome numbers are *x* = 7, *x* = 8, and *x* = 11 [18,57]. Currently, *x* = 7 is considered to be the original primary chromosome number in the genus *Salvia*. The chromosome number *x* = 7 predominates in the Mediterranean region and Southwest Asia [59,69,70], and *x* = 8 is also distributed in East Asia [71]. The basic number *x* = 11 is mainly observed in species from Europe, Russia, and South and Central America [69,72,73]. It is believed that during karyotype evolution, other basic numbers arose from the number *x* = 7 through genomic mutations (e.g., dysploidy, aneupoidy, and polyploidy) [18,70]. Polyploidy is one of the evolutionary strategies of species within the subg. *Calosphace* in South America. The distribution of the species from this subgenus, which occurred from the center of their origin (Central America) to South America, correlates with their ploidy levels [18,73]. High ploidy levels are common in South America, with 34.48% of polyploidy occurring in Argentinean and Mexican species belonging to the subg. *Calosphace* [18]. The chromosome number *x* = 16 could be considered as a polyploid level with the basic number *x* = 8 [18,73].

It is believed that *x* = 6 and *x* = 9 were formed through aneuploid changes in the chromosome number. Dysploidy and dibasic polyploidy were likely involved in the evolution of the secondary base numbers *x* = 11 and *x* = 10 [18]. The basic chromosome numbers *x* = 13 and *x* = 15 could be the result of interspecific hybridization of species having different numbers of chromosomes, with subsequent doubling of chromosomes. For example, *x* = 13 could be dibasic, formed by the combination of *x* = 6 and *x* = 7 [73]. This type of speciation is typical for many flowering plants [74].

The species from the same subgenus might have a common number of chromosomes, as is observed in the subg. *Glutinaria* (Table 2). All species of this subgenus have the same basic chromosome number *x* = 8 [75,76,77,78,79], and this karyological characteristic has been proposed as a diagnostic feature of *Glutinaria* [16]. The basic chromosome number *x* = 8 could be considered as an additional synapomorphy feature of this subgenus. Polyploidy was revealed in several species from the section *Eurysphace*, with both diploidy and tetraploidy observed in *S. przewalskii* Maxim. and *S. evansiana* [72,75,76,77,78].

At the same time, species that belong to different sections of the subgenus may have different chromosome numbers. For example, in the subgenera *Salvia* and *Sclarea*, chromosome number variations are observed both between and within sections (Table 3 and Table 4). This may be a result of intraspecific dysploidy and/or incorrect counting of small chromosomes in a karyotype. Moreover, misidentification of the species identity cannot be ruled out, especially in highly polymorphic species, and therefore, further studies of karyotypes of such *Salvia* species are needed to clarify their basic chromosome numbers.

In species of the subg. *Salvia*, the most common basic chromosome numbers are *x* = 7 and *x* = 8. The chromosome numbers *x* = 9 (in *S. aucheri* subsp. *canescens*) and *x* = 6 (in *S. ringens*) are less common. In species from the sections *Holochilus* and *Hymenosphace* in the subg. *Salvia*, polyploidy was revealed. It is believed that in *S. multicaulis* (sect. *Hymenosphace*), the polyploidy could be a result of the evolution of this species based on the chromosome numbers *x* = 7 (2*n* = 4*x* = 28) and *x* = 8 (2*n* = 4*x* = 32) and aneuploidy (2*n* = (4*x* − 2) = 30) [18]. In *S. bucharica* (sect. *Holochilus*), tetraploidy (2*n* = 4*x* = 32) was found [19]. The karyotype of *S. officinalis*, which is the most famous medicinal species of the subg. *Salvia* (section *Salvia*), includes a diploid chromosome number of 2*n* = 2*x* = 14 [69,72,79]. Furthermore, in some karyotypes of *S. officinalis*, additional B chromosomes were revealed [22,70].

In the subg. *Sclarea*, the basic chromosome numbers vary from 7 to 11 (*x* = 7, 8, 9 10, and 11) (Table 4). In most species from the section *Aethiopis*, *x* = 11 and *x* = 10 were revealed. The species with the basic chromosome numbers *x* = 9 (*S. verbascifolia*) and *x* = 8 (*S. fominii)* are less common. The karyotype of the most economically valuable species, *S. sclarea* (section *Aethiopis*), contains 2*n* = 2*x* = 22 chromosomes [85,86,87]. In this section, examples of polyploidy were revealed in *S. desoleana* and *S. ceratophylla.* In the karyotype of *S. viridis* (monotypic section *Horminum*), different authors determined the same number of chromosomes (2*n* = 2*x* = 16). The section *Plethiosphace* is represented by species with all of the basic chromosome numbers revealed in the subg.*Sclarea*. In some species of the section *Plethiosphace*, polyploidy was also observed. Polyploidy, in combination with aneuploidy, is thought to be an important mechanism of the chromosome number evolution in this section [18].

**Table 4:** Chromosome numbers in *Salvia* species from different sections of the subgenus *Sclarea*.

Sections and Species	Chromosome Number	Source	DNA Content, pg/1C
Sect. *Plethiosphace*			
*S. nemorosa*	2*n* = 2*x* = 14	[70,72,83]	0.56–0.6 [19] 0.55 [81]
*S. tesquicola* (*S. nemorosa* subsp.* tesquicola*)	2*n* = 2*x* = 14	[67]	
*S. algeriensis*	*x* = 7, 9 2*n* = 4*x* = 36 2*n* = 38 2*n* = 6*x* = 42	[61]	
*S. amplexicaulis*	2*n* = 2*x* = 20	[83]	0.74 [19]
*S. austriaca*	2*n* = 2*x* = 18	[72,83]	
*S. deserta*	2*n* = 2*x* = 14, 16	[67]	0.56 [19]
*S. nutans*	2*n* = 2*x* = 22	[67]	0.50 [19]
*S. pratensis*	2*n* = 16, 18, 32	[67]	0.56 [19] 0.46 [81]
*S. transsylvanica*	2*n* = 2*x* = 16	[61]	
*S. verbenaca*	2*n* = 14, 16, 42, 54, 56, 60, 62, 64	[67]	0.48–0.49 [19]
*S. dumetorum*	2*n* = 2*x* = 14	[72]	0.60 [19]
*S. jurisicii*	2*n* = 2*x* = 22	[61,67]	
*S. grandifolia*	2*n* = 4*x* = 40	[88]	
Sect. *Aethiopis*			
*S. aethiopis*	2*n* = 2*x* = 22	[67]	1.50 [19]
*S. sclarea*	2*n* = 2*x* = 22	[67]	0.66–0.69 [19] 0.58 [81]
*S. verbascifolia*	2*n* = 2*x* = 16, 18, 22	[67,72]	
*S. karabachensis*	2*n* = 2*x* = 20	[67]	
*S. argentea*	2*n* = 2*x* = 20, 22	[59,72,83]	
*S. frigida*	2*n* = 2*x* = 20	[59]	
*S. fominii*	2*n* = 16	[67]	
*S. palestina*	2*n* = 2*x* = 20	[59,67]	
*S. poculata*	2*n* = 2*x* = 20	[59]	
*S. limbata*	2*n* = 2*x* = 22	[59]	0.86 [80]
*S. desoleana*	2*n* = 4*x* = 44	[67]	0.78 [19]
Sect. *Horminum*			
*S. viridis*	2*n* = 2*x* = 16	[70,72,83]	0.53 [19] 0.43 [81]

The DNA content of species is an important feature for understanding the evolution of their genomes, which is also necessary for the genome sequencing process [89]. In the Plant DNA C-values Database, there are rather limited data on DNA content in *Salvia* species [81]. In 2022, the DNA content of many *Salvia* species was measured using flow cytometry [19]. However, *Salvia* genome sizes did not always correspond to the basic chromosome numbers, and the DNA content might not be an indicator of the polyploidy level of the specimens (Table 1, Table 2 and Table 3) [19].

Classical methods of monochrome chromosome staining showed that *Salvia* karyotypes contained small-sized chromosomes (0.3–5 µm). Based on the centromere index, karyograms and idiograms of various *Salvia* species were constructed [57,60,61,78,87,90,91]. However, the morphology of these chromosomes varies greatly depending on the degree of their compaction, which affects the final formula of the karyotype. For example, the morphology (the length, centromere position, and arm index) of small *S. sclarea* chromosomes has differed in various studies, which has led to different versions of this species karyotype formula [85,86,87]. For *S. officinalis* and *S. glutinosa*, karyotype formulas were also demonstrated, although pairs for most chromosomes could not be clearly determined [57,61]. Karyotype analyses in five populations of *S. miltiorrhiza* resulted in two karyotype formulas of this species (2*n *= 2*x* = 16 = 8m + 8sm and 2*n* = 2*x* = 16 = 6m + 10 sm). Moreover, since the chromosomes were studied in different degrees of compaction, they were not accurately identified [82].

Thus, the small sizes of *Salvia* chromosomes have made it difficult to determine their morphological features (centromere index, presence of secondary constrictions, etc.). However, modern methods of molecular cytogenetics have not been used for the analysis of *Salvia* karyotypes until recently, which has prevented accurate karyotype characterization even in the most studied *Salvia* species. At the same time, various tandem DNA repeats are currently widely used to study the chromosomal organization of plant genomes, intra- and interspecific chromosomal variability, and the evolution of plants [92,93,94].

### 3.2. Integration of the Repeatomic and Cytogenomic Data

Repetitive DNA sequences make up to 90% of the total DNA [95,96,97,98]. The plant repeatome comprises highly abundant transposable elements and tandem repeats (including ribosomal genes and satellite DNA), which are diverse components of the genome [96,99,100,101]. These elements are involved in genome organization and evolution, as they can change their location and copy number, leading to changes in gene expression and regulatory networks [96,97,99,101,102,103,104]. Transposable elements are subdivided into class I (retrotransposons, including LTR retrotransposons) and class II (DNA transposons) according to the structural characteristics and mode of replication [99,100,101]. LTR retrotransposons, including the Ty1-Copia and Ty3-Gypsy superfamilies, are very common and make up to 85% of plant nuclear DNA [101,103,104,105]. LTR retrotransposons are considered to be major contributors to plant genome changes [98,103,104]. The high redundancy of these retroelements occurs because of their ability to replicate and generate new copies of themselves via the ‘copy and paste’ mechanism (opposed to the ‘cut and paste’ mechanism of transposons) and thus increase the genome size. At the same time, LTR copies can be eliminated through both solo LTR formation and accumulation of deletions, which reduce the genome size [102]. The LTR mobility mechanism participates in the production of RNA molecules, which are retrotranscribed into DNA and then inserted into new sites of the host genome [102]. Currently, LTR retrotransposons are actively studied in plants as molecular markers and as factors contributing to new phenotype markers. In particular, they are used in the study of plant evolution, diversity, and chromosome structure [21,96,97,100,105].

In eukaryotic genomes, ribosomal DNA (rDNA) is an important constituent involved in the protein synthesis processes [106,107]. It comprises two main classes of genes: 45S rDNA, encoding 26S (25.5S), 5.8S, and 18S rRNA, and 5S rDNA, encoding 5S rRNA. Ribosomal DNA sequences are tandemly arranged, relatively conserved in their structure, and often used in FISH (fluorescence in situ hybridization) assays, as well as in phylogenetic studies as synapomorphic features [22,97,107,108].

Satellite DNA families (satDNAs) are highly repetitive DNA sequences organized in tandem arrays up to 100 Mb in size. The identity of sequences in the array is achieved through the process called “concerted evolution”, which results in the maintenance of homogeneity of satDNA monomers within a species during evolution [109,110]. An example of this process is the CON1/CON2 satellite families, which are highly conserved in closely related cereal species [111]. At the same time, satDNAs are rapidly evolving fractions of plant genome that can vary in their abundance and distribution in genomes. Furthermore, satDNAs can originate from transposable elements (transposons and retrotransposons). These mobile elements can facilitate not only satDNA formation but the spread of satDNAs in the genome [100,103,112]. It has been shown that satDNAs might differ in copy number and nucleotide composition (even between related species and generations), resulting in high polymorphism in the length of satellite arrays [113]. Some satDNA sequences, however, can be relatively conserved during long evolutionary periods, which is probably due to their interaction with heterochromatin-associated proteins, as well as their possible regulatory role in gene expression [109,110]. It is considered that the evolution of species-specific satDNAs could be a result of copy number changes in a library of satellite sequences common to a group of species [103,109,110,114].

**Figure 2:**
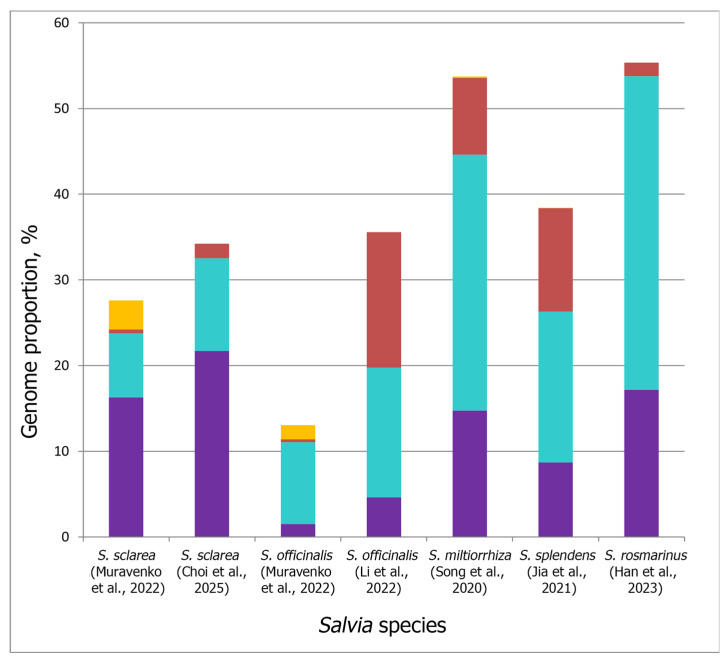
Genome proportions of most abundant DNA repeats (Ty1-Copia (purple), Ty3-Gypsy (aqua), DNA transposons (red), and satellite DNA (orange) in several *Salvia* species according to different studies [21,22,24,25,27,28].

The analysis of the repeatome composition of several *Salvia* species was performed based on high-throughput sequencing data using different bioinformatic approaches including RepeatExplorer2 and TAREAN [22]; RepeatModeler (v2.0.1)24 and RepeatMasker (v4.1.2-pl)25 [28]; LTR-Finder (v1.0.2), MITE, RepeatScout (v1.0.5) and PILER (v1.0) [21]; RepeatModeler v1.0.10, RECON v1.08, and RepeatScout v1.0.5 [24]; and Itrharvest and LTR_FINDER_parallel [25]. As a result, genome proportions of most abundant DNA repeats were revealed in several *Salvia* species (Figure 2).

It was shown that genome proportions of the repeated DNA sequences could differ between *Salvia* species, and retrotransposons made up the majority of their repeatomes [21,22,24,25,27,28]. At the same time, the repeatomes of these species contained different number of retrotransposons, which could contribute to the genome diversity and evolution of the species [96,97,103,105]. For example, in the *S. miltiorrhiza* genome (subg. *Glutinaria*), various LTR retrotransposon families were analyzed. The revealed high percentage of young LTR retroelements indicated their activity in very recent evolutionary history. It was suggested that the genome of this species experienced multiple amplification bursts of LTR elements within the last 1 Mya. These young LTR retrotransposons could contribute to the increase in the genome size in *S. miltiorrhiza* by more than 10% (65 Mb) (based on an average size of 6 kb per LTR elements). Moreover, the analysis of the evolutionary divergence of genome of this species indicated a possible recent species-specific whole-genome duplication in *S. miltiorrhiza*, and this event was probably followed by rapid fragmentation and further dynamic changes in genome structure [21].

The proportion of DNA transposons in the repeatomes of *S. miltiorrhiza* (subg.*Glutinaria*) and *S. splendens* (subg. *Calosphace*) is significantly higher than that in the repeatomes of *S. sclarea* and *S. rosemarinus*. Satellite DNA in the genome of *S. splendens* makes up only 0.01%, which is less than the proportion of satDNA in *S. officinalis* and *S. sclarea* (1.63% and 3.4%, respectively). SatDNA and transposable elements are predominantly located in the heterochromatic regions, and these repetitive elements can interact with each other. Moreover, satDNA can be formed from different types of transposable elements and/or their structural components, although the reverse process is also possible [103,112]. In addition, differences in repeatome composition were revealed in different specimens of the same species (e.g., *S. sclarea* and *S. officinalis*) (Figure 2), which could be related to the specifics of the methods used. However, the influence of intraspecific diversity cannot be ruled out [22,27,28].

The analysis of the *Salvia* repeatomes opened up new possibilities for using different tandem DNA repeats as molecular chromosomal markers in FISH assays. FISH mapping of ribosomal genes was performed on the chromosomes of *S. miltiorrhiza* (2n = 16) (subg. *Glutinaria*, section *Drymosphace*) [20,21]. Liu et al. [20] revealed 45S rDNA clusters on five chromosomes in the karyotype of *S. miltiorrhiza*. At the same time, Song et al. [21] observed 45S rDNA clusters on two chromosome pairs (LG1 and LG4) and detected 5S rDNA on two other chromosome pairs (LG2 and LG8) in *S. miltiorrhiza*. Variability in minor 45S rDNA loci was also revealed in *S. officinalis* [115]; this phenomenon is widespread in plants [97,108,116]

**Figure 3:**
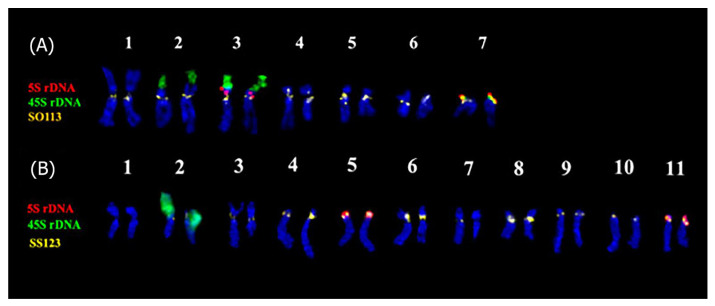
The karyotypes of *Salvia officinalis* (**A**) and *Salvia sclarea* (**B**) demonstrating chromosome localization of 5S rDNA, 45S rDNA, and the marker satDNAs probes. The probe names and their preudocolors are shown on the left. The figure is adapted from Muravenko et al. [22].

**Figure 4:**
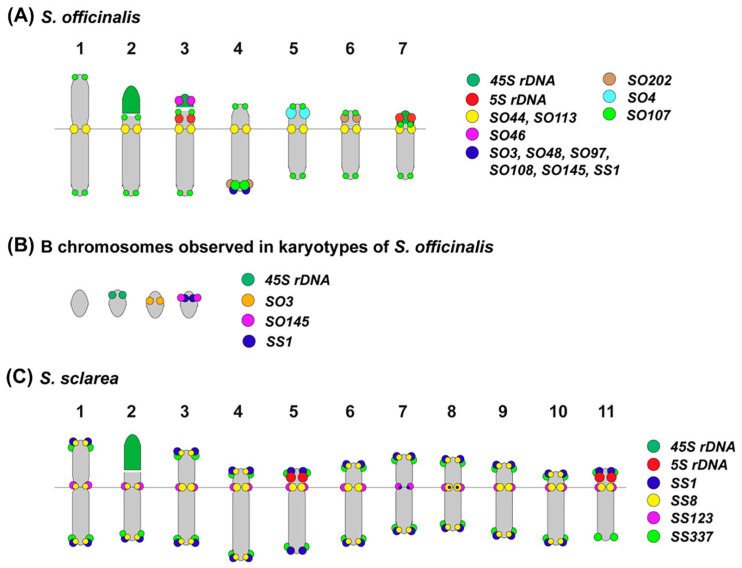
Chromosome schematic representation of *Salvia officinalis* (**A**), the revealed B chromosomes (**B**), and *Salvia sclarea* (**C**) demonstrating the positions of 5S rDNA, 45S rDNA, and the oligonucleotide-based satDNA probes. The probe names and their preudocolors are shown on the right. The figure is adapted from Muravenko et al. [22].

In a recent study, a comparative analysis of the karyotypes of *S. officinalis* and *S. sclarea* using FISH with 45S rDNA, 5S rDNA, and various satDNAs was carried out to assess the intra- and interspecific diversity of their genomes (Figure 3) [22]. In both species, 5S rDNA loci were observed on two chromosome pairs (on pairs 3 and 7 in *S. officinalis* and on pairs 5 and 11 in *S. sclarea*); 45S rDNA clusters were localized on chromosome pair 2 in the karyotype of *S. sclarea* and on three chromosome pairs (2, 3, and 7) in *S. officinalis*, and two of them (3 and 4) were the satellite chromosomes (Figure 3 and Figure 4) [22]. However, a previous karyotype analysis of this species, based on classical methods of monochrome chromosome staining, did not reveal these satellite chromosomes [85,86,87].

A repeatome study based on high-throughput sequencing followed by bioinformatics analyses identified eight high-confidence putative satDNAs and four low-confidence putative satellites in *S. officinalis*. In *S. sclarea*, three high-confidence putative satDNAs and one low-confidence putative satDNA were identified. These satDNAs were FISH-mapped on chromosomes of these species (Figure 2 and Figure 3). The results showed that in *S. officinalis* and *S. sclarea*, almost all satDNAs were localized in clusters in the pericentromeric and distal regions of chromosomes [22]. Moreover, in *S. officinalis*, the majority of satDNAs were localized in the long arm of one chromosome pair, 4 (Figure 4), which is not typical for plants [117,118,119,120]. This could be related to the specific distribution of these repeats in the genome of this species.

Localization of some satDNAs made it possible to refine the identification of chromosome pairs (Figure 3 and Figure 4) in the karyotypes of both species, demonstrating their suitability as chromosomal markers [22]. In addition, the use of satDNAs with variable localization of minor sites on chromosomes [22] made it possible to begin studying intraspecific chromosomal variability in *S. officinalis* and to show that the karyotype of this species contains at least two variants of chromosomes 4, 5, and 6, as well as several variants of B chromosomes [115].

The identification of common satDNAs in the genomes of *S. officinalis* and *S. sclarea* confirmed their common origin as well as the possibility of using the identified marker satDNAs in further karyotype studies in related species from both subgenera [22,48,49].

At the same time, for other *Salvia* species, FISH mapping of 45S rDNA, 5S rDNA, and satDNAs has not yet been performed, and their karyotype structure remains insufficiently studied. Further research on *Salvia* karyotypes is needed using integrated approaches based on whole-genome sequencing data and FISH chromosome mapping of various DNA repeats as chromosomal markers. Such studies would open up opportunities for investigating the chromosomal organization and variability of karyotypes of the individual species. Moreover, integration of cytogenetic and genomic data is very important for resolving conflict issues in *Salvia* taxonomy and phylogeny. Thus, despite advances in sequencing technology, further cytogenetic studies are required to characterize *Salvia* genomes.

## 4. Conclusions

The studies presented in this review have significantly increased our understanding of the genetic structure and evolutionary biology of the genus *Salvia*. Research into the medicinal properties of different *Salvia* species has expanded significantly in recent decades, which requires a deeper understanding of the taxonomy, genetics, genomics, and phylogeny of *Salvia* species and their hybrids. Moreover, comprehensive studies are very important for conservation of species, including valuable hybrids, and their population diversity. Further research in these fields will contribute to the improvement of the taxonomy of the genus *Salvia* and understanding of the pathways of evolution of genomes, chromosomes, and useful genes, which will increase the efficiency of breeding of new *Salvia* varieties and intraspecific hybrids with valuable biochemical compositions.

## Figures and Tables

**Table 1:** Subgeneric classification of the genus *Salvia* (adopted from Kriebel et al. [2]).

Subgenus (Subgeneric Classification of *Salvia* [2]	Estimated Number of Species	Clade Number According to Will and Claßen-Bockhoff [9]	Staminal Type According to Walker and Sytsma [39]
*Salvia* L.	70	Clade I	A
*Sclarea* (Moench) Benth.	120	Clade I	B
“*Heterosphace*”	43	Clade I	A
*Rosmarinus* (L.)	3		C
*Perovskia* (Kar.)	8		D
*Calosphace* (Benth.) Epling	550	Clade II	E, F, G
*Audibertia* (Benth.)	19	Clade II	H, I
*Meriandra* (Benth.)	2		J
*Dorystaechas* (Boiss. & Heldr. ex Benth.)	1		K
*Zhumeria* (Rech. f. & Wendelbo)	31	Clade III (in part)	L, M
*Glutinaria* (Raf.)	100	Clade IV	N

**Table 2:** Chromosome numbers in the *Salvia* species from different sections of the subgenus *Glutinaria*.

Sections and Species	Chromosome Number	Sources	DNA Content, pg/1C
Sect. *Sonchifoliae*	No data		
Sect. *Notiosphace*			
*S. plebeia*	2*n* = 2*x* = 16 + 0–2B, 32	[67]	
Sect. *Substoloniferae*			
*S. trijuga*	2*n* = 2*x* = 16	[75,78]	0.42 [19]
Sect. *Glutinaria*			
*S. glutinosa*	2*n* = 2*x* = 16	[61,80]	0.83 [80] 1.07 [81]
*S. nubicola*	2*n* = 2*x* = 16	[67]	1.11 [19]
*S. nipponica*	2*n* = 2*x* = 16	[67]	1.21 [19]
Sect. *Annuae*			
*S. roborowskii*	2*n* = 2*x* = 16	[75,78]	
Sect. *Eurysphace*			
*S. campanulata*	*n* = 8	[67]	
	2*n *= 4*x* = 32	[78]	
*S. przewalskii*	2*n* = 2*x* = 16	[72]	
	2*n *= 4*x* = 32	[71,75,78]	
*S. evansiana*	2*n* = 2*x* = 16	[78]	
	2*n *= 4*x* = 32	[71,76]	
Sect. *Drymosphace*			
*S. miltiorrhiza*	2*n* = 2*x* = 16	[71,75,76,82]	0.7 [19] 0.6 [21] 2.05 [81]
Sect. *Sobiso*			
*S. pygmaea*	2*n* = 2*x* = 16	[67]	

**Table 3:** Chromosome numbers in *Salvia* species from different sections of the subgenus *Salvia*.

Sections and Species	Chromosome Number	Sources	DNA Content, pg/1C
Sect. *Salvia*			
*S. officinalis*	2*n* = 2*x* = 14	[59,72,79]	0.59 [19] 0.49 [81]
	2*n* = 2*x* = 14 + 1–2B	[70]	
*S. fruticosa*	2*n* = 2*x* = 14	[59]	0.58 [19] 0.84 [81]
*S. grandiflora*	2*n* = 2*x* = 14, 16	[72,83]	
*S. scabiosifolia*	2*n* = 2*x* = 14	[83]	0.77 [19]
*S. aucheri* subsp. *canescens*	2*n* = 2*x* = 18	[59]	
*S. ri* *n* *ge* *n* *s*	2*n* = 2*x* = 12, 16	[67]	0.61 [81]
*Sect. Holochilus*			
*S. bucharica*	2*n* = 4*x* = 32	[19]	1.22 [19]
*Sect. Hyme* *n* *osphace*			
*S. pomifera*	2*n* = 2*x* = 14	[59,70]	
*S. hydrangea*	2*n* = 2*x* = 14	[59,70]	
*S. multicaulis*	2*n* = 4*x* = 28	[59]	1.13 [81]
	2*n* = 16, 32, 30	[70,84]	

## Data Availability

All data generated or analyzed during this study are contained within the article.
